# Effect of Target Power on Microstructure, Tribological Performance and Biocompatibility of Magnetron Sputtered Amorphous Carbon Coatings

**DOI:** 10.3390/ma16175788

**Published:** 2023-08-24

**Authors:** Vishnu Shankar Dhandapani, Ramesh Subbiah, Elangovan Thangavel, Chang-Lae Kim, Kyoung-Mo Kang, Veeravazhuthi Veeraraghavan, Kwideok Park, Dae-Eun Kim, Dongkyou Park, Byungki Kim

**Affiliations:** 1 Department of Electromechanical Convergence Engineering, Korea University of Technology and Education, Cheonan 31253, Republic of Korea; 2 School of Mechatronics Engineering, Korea University of Technology and Education, Cheonan 31253, Republic of Korea; 3 Center for Biomaterials, Korea Institute of Science and Technology (KIST), Seoul 02792, Republic of Korea; 4 Department of Biomedical Engineering, Korea University of Science and Technology (UST), Daejon 34113, Republic of Korea; 5 Smart Energy Material Laboratory (SEML), Department of Energy Science and Technology, Periyar University, Salem 636011, India; 6 Department of Mechanical Engineering, Chosun University, Gwangiu 61452, Republic of Korea; 7 Department of Mechanical Engineering, Yonsei University, Seoul 03722, Republic of Korea; 8 PG & Research Department of Physics, PSG College of Arts & Science, Coimbatore 641014, India

**Keywords:** amorphous carbon, tribological property, preosteoblasts, wettability, friction simulation, microstructure

## Abstract

The tribological properties and preosteoblast behavior of an RF magnetron-sputtered amorphous carbon coating on a Si (100) substrate were evaluated. The graphite target power was varied from 200 to 500 W to obtain various coating structures. The amorphous nature of the coatings was confirmed via Raman analysis. The contact angle also increased from 58º to 103º, which confirmed the transformation of the a-C surface from a hydrophilic to hydrophobic nature with an increasing graphite target power. A minimum wear rate of about 4.73 × 10^−8^ mm^3^/N*mm was obtained for an a-C coating deposited at a 300 W target power. The 300 W and 400 W target power coatings possessed good tribological properties, and the 500 W coating possessed better cell viability and adhesion on the substrate. The results suggest that the microstructure, wettability, tribological behavior and biocompatibility of the a-C coating were highly dependent on the target power of the graphite. A Finite Element Analysis (FEA) showed a considerable increase in the Von Mises stress as the mesh size decreased. Considering both the cell viability and tribological properties, the 400 W target power coating was identified to have the best tribological property as well as biocompatibility.

## 1. Introduction

Carbon is a unique material that can be prepared in various forms such as graphite, graphene, graphene oxide, carbon nano tubes (CNT), hydrogenated amorphous carbon (a-C:H), hydrogen-free amorphous carbon (a-C) and diamond-like carbon (DLC) [1,2,3,4,5,6,7]. For the past few decades, because of their good mechanical properties and chemical stability, these materials have been widely utilized and explored as surface protective coatings in various applications [5,6,7,8,9]. Much attention has been devoted to determining the optimum coating method since coating parameters and conditions play a vital role in deciding the physical and chemical properties of the coating. In this regard, the coating method significantly affects the tribological properties as well as the biocompatibility of the coating. For instance, it has been shown by various researchers that the thickness of an a-C coating should be optimized with respect to the residual stress, which can negatively affect the tribological properties if it is not properly controlled [9,10,11,12]. Hence, it is important to optimize the coating conditions and study its physical and chemical properties to enhance the quality of the coating towards its applications [13,14,15].

Extensive research has been conducted to identify the effects of various process parameters on the resulting coating properties. Afshar et al. deposited an a-C coating by varying its substrate temperature and reported that the physical properties of coatings were highly dependent on the substrate temperature as well [16]. Savhn et al. reported that the roughness of an a-C:Cr coating has much influence on its friction and wear properties, whereas there was no such notable influence in the case of a-C:W coatings [17]. Liu et al. [18] reported the frictional, scratch and wear resistance properties of an a-C coating by varying its thickness, and groups have also reported the importance of the thickness of a-C coatings in their tribological behavior [19,20].

The biocompatibility of carbon-based coatings has also been a topic of great interest for their utilization in biomedical applications [10,21,22,23]. Several studies reported the improvement of preosteoblast viability, which is an important phenomenon for bone tissue regeneration, after the surface modification of metals used for implants with carbon coatings. Subbiah et al. reported the enhanced cell viability and osteogenic differentiation of preosteoblasts on graphene-oxide-coated titanium substrates [24]. The improved preosteoblast cell proliferation and cell viability of titanium-containing a-C coatings on 316L SS substrates were also reported in our earlier work [25]. Rodil et al. reported enhanced biocompatibility via in vitro studies on different carbon-based coatings (a-C, a-C:N and a-C:H) on 316L SS substrates, in which pure a-C film showed particularly better cell viability than the other a-C based coatings [26]. Similarly, several other reports also demonstrated the improved biocompatibility of metal implants after surface modification with carbon-based coatings [27,28,29,30,31]. From a survey of the literature, it was evident that various parameters including the substrate temperature, thickness, chemical composition, roughness, etc., all have significant influences on the mechanical and tribological properties and biocompatibility of a-C coatings. However, in most previous studies, the effect of the coating parameter was investigated with respect to either the tribological properties or the biocompatibility of the coating. The findings of such independent investigations have limited value since in actual biomedical application, both properties are relevant. In other words, for actual application, the coating should have adequate biocompatibility with sufficient durability, which can be inferred from its tribological properties. Therefore, there is a need to optimize both of these properties together for a given coating system.

In this work, both the tribological properties and biocompatibility of a-C coatings deposited on Si (100) substrates were assessed and optimized with respect to the power input of the deposition process. The samples were prepared via the RF magnetron sputtering process using different target powers to create a-C coatings with varying properties. The coatings were characterized using Raman spectroscopy, atomic force microscopy (AFM) and a contact angle measurement system. In order to analyze the frictional behavior and wear resistance of these coatings, sliding tests were performed. The effect of the mesh size on the von Mises stress was analyzed using an Abaqus simulation. Furthermore, the biocompatibility and preosteoblast behavior on the a-C-coated samples were determined through a cell proliferation test as well as live and dead cell assay analyses.

## 2. Materials and Methods

### 2.1. Coating Procedure

The coating conditions were the same as those of our earlier reports [25,31], and the condition of the coatings were as follows: Graphite target was used with different target powers (200, 300, 400 and 500 Watts) via RF magnetron sputtering to deposit a-C coatings on silicon (100) substrates. A base pressure of 0.003 Pa (3 × 10^−5^ mbar) and a working pressure of ~0.75 Pa (7.5 × 10^−3^ mbar) for the deposition chamber was maintained. High purity argon (99.999%) with a flow rate of 50 sccm was sustained to the chamber using MKS mass flow controllers. Deposition time was adjusted to obtain a constant thickness of about 70 nm (±3 nm). A stylus profiler (Bruker’s DektaXT^TM^) was used to measure the thickness of the a-C coatings. A step was created on the substrate by masking it before placing it for deposition of a-C coating in the vacuum chamber, and the step was then measured using the stylus profilometer to measure the thickness of the coating. No substrate bias was applied during the deposition process. The substrate holder was rotated at 7 rpm.

### 2.2. Tribological Test

The tribological properties followed those of our earlier reports [25,31]. A custom-built reciprocating tribo-tester was used to investigate the friction and wear properties of deposited a-C coatings. The test conditions are given in Table 1. The data acquisition system was used to record the co-efficient of friction (COF) and the number of sliding cycles automatically. Tests were repeated five times with each specimen to obtain an average friction value. Wear rate of the coatings were calculated using the wear equation proposed by Archard [32].

### 2.3. Finite Element Analysis

To accurately simulate the sliding condition of the optimized 400 W coating, the thickness (70 nm) and average friction coefficient (0.45) were incorporated into the modeling from the experimental data. Since the actual thickness of the coating was too low (70 nm) to obtain the Young’s modulus without substrate influence, this value was taken from our earlier report in which the coating was deposited in similar conditions with a thickness of 200 nm [31]. The properties such as the Young’s modulus [33,34] and Poisson’s ratio [35,36] of the Al_2_O_3_ tip and silicon substrate were taken from earlier reports. After wear generation, the silicon substrate will exhibit high friction [37], which may lead to high wear generation and high von Mises stress during simulation. Since analyzing the substrate is not the main focus of this work, the simulation of the silicon substrate was avoided. The main focus of this simulation was to study the influence of mesh size on von-Mises stress during sliding, so the mesh sizes of 0.05 mm and 0.025 mm and gradient mesh were used in this work. Therefore, only the 400 W coating was taken for simulation modeling. A portion of the substrate was used in modeling to save computation time, but it was sufficiently large to avoid boundary effects during the simulation. Similarly, for the gradient mesh, the portion of the substrate and the tip size were further reduced in modeling compared to the 0.025 mm and 0.05 mm modeling, but it was kept sufficiently large to avoid boundary effects. The three steps involved in the simulation process are the establishment of contact, the adjustment of the normal load to 10 mN and sliding the tip against the coating surface. 

### 2.4. Cell Behavior

The viability, proliferation and morphology of the cells were determined as previously described [24,25]. Briefly, 20,000 cells (preosteoblasts, MC3T3-E4, ATCC, Manassas and VA) were seeded on 1 cm × 1 cm uncoated silicon substrates and a-C coated silicon substrates for various target powers (200 W, 300 W, 400 W and 500 W). The samples were incubated in α-minimum essential medium (α-MEM) with 10% fetal bovine serum (FBS) and 1% penicillin/streptomycin under standard culture conditions (37 °C and 5% CO_2_). The medium was removed on day 3, and the samples were incubated with a mixture of phosphate-buffered saline (1× PBS) (1 mL), 2 μM calcein AM and 4 μM ethidium homodimer at room temperature for 15 min. Then, the samples were washed in 1× PBS, and the live and dead cells were imaged on a fluorescence microscope (CKX41-F32FL; Olympus). In addition, the cell proliferation rate was tested using a cell counting kit-8 (CCK-8; Dojindo, Japan). Briefly, on day 3, CCK-8 solution was added to the cell-cultured samples (final concentration of 10% *v*/*v*) and incubated at 37 °C for 2 h. The absorbance of the resulting solution was measured at 450 nm using a Multiscan microplate reader (Thermo Scientific, Rockford, IL, USA). To determine whether the cell morphology varied as a function of the surface coating, the cells were examined in the scanning electron microscope. Briefly, after 3 days of culture as described above, the cells were washed in 1× PBS, fixed in 4% paraformaldehyde, dehydrated in graded ethanol solution (50–100%), dried, sputter-coated with platinum and imaged using SEM (Phenom G2 pro desktop, Eindhoven, the Netherlands). The cell behavior results are presented as mean ± standard deviation (n = 3) and are representative of three independent experiments. Data were analyzed via one-way analysis of variance (ANOVA) and Tukey’s post hoc test to evaluate statistical differences among the samples using GraphPad Prism 5 (GraphPad Software Inc., La Jolla, CA, USA).

## 3. Results

### 3.1. Raman Analysis

**Figure 1:**
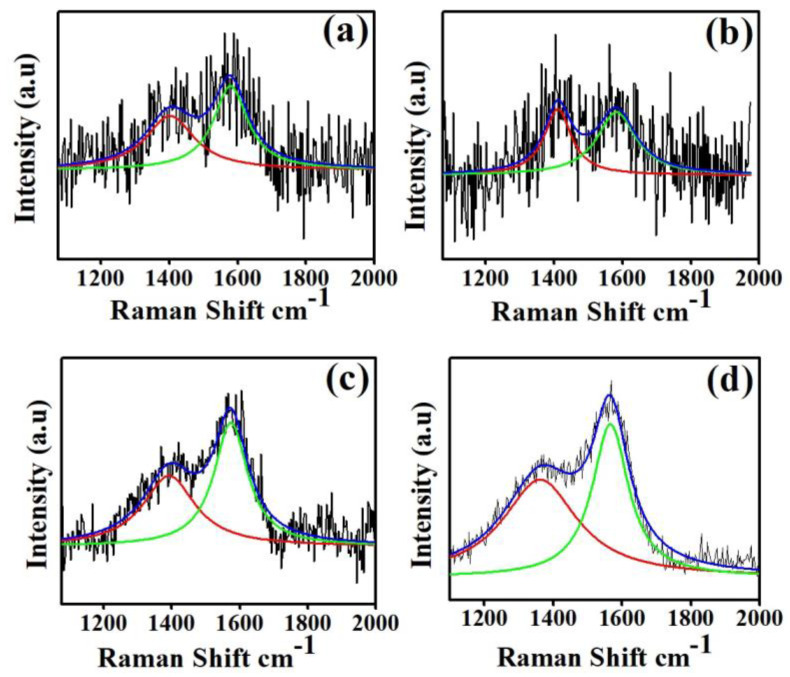
Raman spectra of (**a**) 200 W, (**b**) 300 W, (**c**) 400 W and (**d**) 500 W coatings.

The Raman spectra were measured using the 514.5 nm line of an argon-ion laser under back-scattering geometry with a liquid-nitrogen-cooled CCD detector (Labram HR) in ambient conditions. Figure 1 shows the Raman spectra of the a-C coatings on Si (100) substrates with different target powers from 200 to 500 W. The D and G bands of the coatings were obtained between 1378–1410 cm^−1^ and 1572–1582 cm^−1^, respectively (Table 2). The D and G band values are given in Table 2; these values were found to be closely matching with previously reported values on amorphous carbon [38,39,40]. The D and G band values were found to decrease with the increasing graphite target power from 200 to 500 W. For the indirect quantitative analysis, *sp^2^* and *sp^3^* were expressed by calculating the ratio of the D peak to the G peak (I_D_/I_G_). The I_D_/I_G_ values of 200, 300, 400 and 500 W a-C coatings were 0.82, 1.03, 0.75 and 0.70, respectively. The I_D_/I_G_ ratio was found to decrease with the increasing sputtering power of graphite, which confirmed the increased *sp^3^* bond formation in the coatings. The lower shift in the wavenumbers of the D and G bands also confirmed the increase in the *sp^3^* bonds in a-C coatings [41]. Wei et al. deposited metal containing an amorphous carbon coating and observed a similar kind of shift in the G band. They postulated that this lower shift was due to the reduced internal stress of the coatings [42]. The results suggest that the graphite target power played an important role in determining the surface properties of the a-C coatings deposited on Si (100) substrates.

### 3.2. Surface Topography

**Figure 2:**
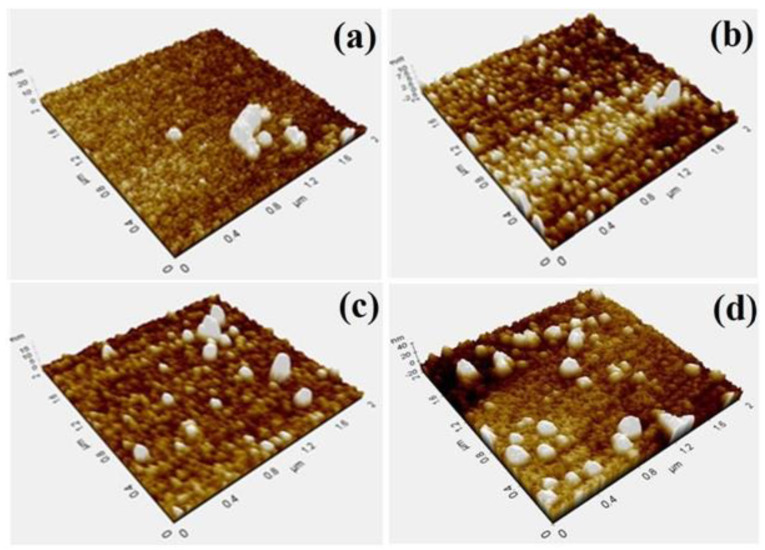
Atomic force microscope (AFM) images of (**a**) 200 W, (**b**) 300 W, (**c**) 400 W and (**d**) 500 W coatings.

An atomic force microscope (Seiko SPA400) was used to measure the surface topographic parameters. The surface topography of a-C coatings with different target powers are shown in Figure 2. The average roughness of the coating was found to increase with the increase in the graphite target power. From the analysis, for the sample with a 200 W target power, the particles were observed to be in random, whereas for the 300 W coatings, an increase in the particle size was observed, and the trend showed an increase in the particle size with an increase in the target power to 400 and 500 W. An increase in the surface roughness with the increasing substrate temperature of the DLC film deposited on the Si (100) substrate via pulsed laser deposition was observed by Asl et al. [43]. The average roughness (Ra) values were about 1.32, 0.99, 1.24 and 3.30 nm for the 200, 300, 400 and 500 W coatings, respectively. The other surface topography parameters, namely the average height (R_z_), the route mean square roughness (R_q_) and the average peak-to-valley roughness (R_pv_), are given in Table 2. The results reveal that the influence of target power on the surface topography of the a-C coatings was significant.

### 3.3. Contact Angle Measurement

**Figure 3:**
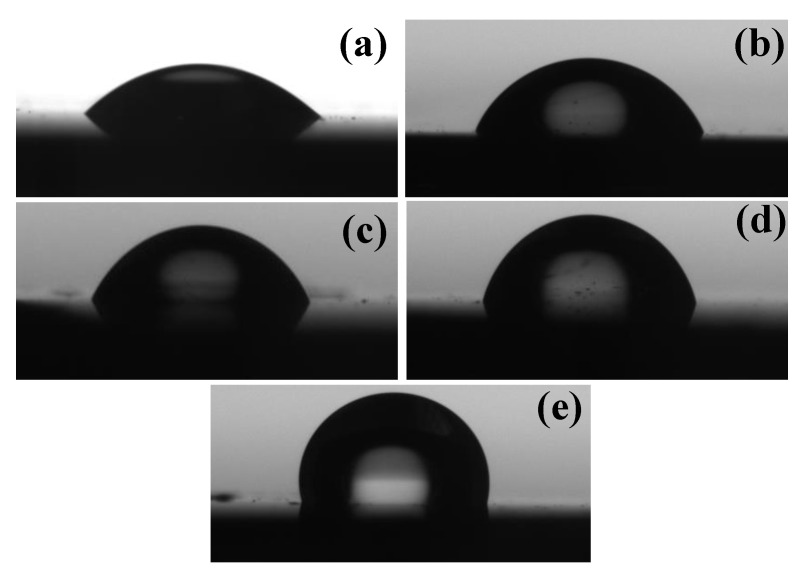
Contact angle measurement of (**a**) uncoated Si and (**b**) 200 W, (**c**) 300 W, (**d**) 400 W and (**e**) 500 W coatings.

A contact angle measurement apparatus (Kriuss DSA 20E) was used to measure the contact angle. Contact angle measurements were carried out to assess the wettability of the coatings. Typically, contact angle values above and below 70° are said to be hydrophobic and hydrophilic, respectively [44]. Figure 3 shows the water contact angle image of the uncoated and a-C-coated Si (100) substrates. The contact angle measurements of bare silicon and the 200, 300, 400 and 500 W a-C coatings were 34.5°, 58°, 63°, 74° and 103°, respectively. The results indicate that the 200 and 300 W coatings exhibit a hydrophilic nature, whereas the 400 and 500 W coatings exhibit a hydrophobic nature. Thus, increasing the graphite target power increased the contact angle, which led to the formation of a hydrophobic surface. In general, the contact angle decreased with the increasing sp^3^ content of the carbon coatings, contrary to our results, where the contact angle increased with the increase in sp^3^ [45]. B.K. Tay et al. reported that the contact angle decreased with the increasing the oxygen exposing time until 7 min, and when it increases to 9 min, the contact angle also increased, which shows more oxygen exposure/contamination after a certain level, led to an increase in the contact angle [46]. Similarly, the increase in the sp^3^ content increased the oxygen contamination more than the sp^2^ content coatings [45], and this may be the reason for the increase in the contact angle with the increasing sp^3^ content in our a-C coating [46]. It was postulated that the increase in the contact angle was due to the reduction in the surface energy of the coatings [43,47].

### 3.4. Tribological Behavior

#### 3.4.1. Friction

**Figure 4:**
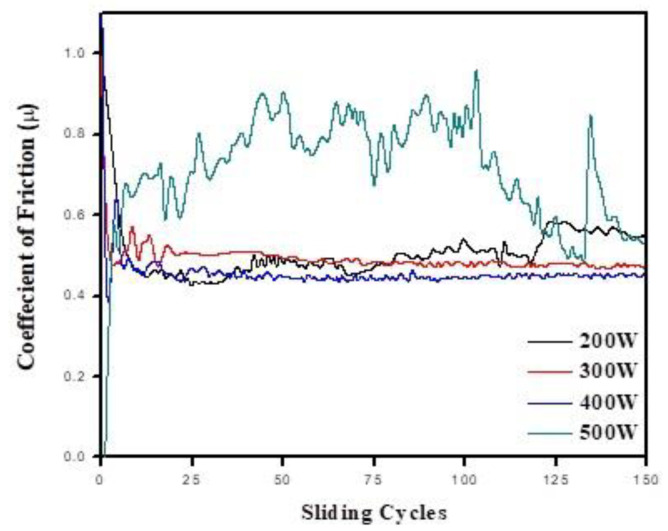
Coefficient of friction of a-C coatings with different target powers.

The frictional behaviors of the coated samples were investigated using a reciprocating tribo-tester. The coefficient of friction (CoF (μ)) values of the 200 W, 300 W, 400 W and 500 W coatings sliding against an alumina (Al_2_O_3_) sphere are given in Table 3. After sliding for 150 cycles, the average COF values were 0.50, 0.49, 0.45 and 0.71 for the 200, 300, 400 and 500 W coatings, respectively. Thus, the average COF value was found to decrease with the increasing graphite target power from 200 to 400 W, and it was found to increase significantly with a further increase in the target power to 500 W. The 200 W coating showed a small fluctuation in the COF while sliding, and the COF value suddenly increased after 120 sliding cycles (Figure 4). Compared to the 200 W coating, the 300 W coating showed a relatively small fluctuation in the COF up to 25 cycles, and after that, it showed no fluctuation until the end of the sliding cycles (Figure 4). The lowest COF value of 0.45 was found for the 400 W coating, and there was no fluctuation in the value during the sliding process. The 500 W coating showed a significantly higher COF value compared to all the other coatings. The high COF of the 500 W coating was attributed to the high surface roughness of the coating [48].

#### 3.4.2. Wear

A 3D laser scanning microscope was used to obtain the wear area by measuring the cross section of the wear track, and the results are given in Table 3. Based on the wear volume obtained from the profile measurements, the wear rates of the coatings were calculated to be 1.35 × 10^−7^, 4.73 × 10^−8^, 5.40 × 10^−8^ and 5.58 × 10^−7^ mm^3^/N*mm for the 200 W, 300 W, 400 W and 500 W coatings, respectively. The wear rate was found to be less for the 300 W and 400 W coatings. Though the 300 W coating has a low wear rate, it was observed that the coating was peeled off from the substrate in some places.

### 3.5. Finite Element Analysis

**Figure 5:**
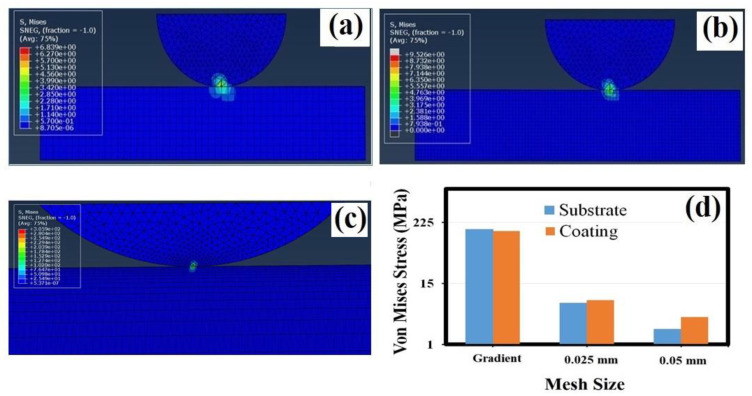
FEA simulation results of (**a**) 0.025 mm mesh size; (**b**) 0.05 mm mesh size; (**c**) gradient mesh and (**d**) von-Mises stress on coating and substrate for all mesh sizes.

A Finite Element Analysis (FEA) using ABAQUS 2022 was performed with different mesh sizes, as shown in Figure 5. Since the aim of this study is to analyze the effect of mesh size on the von Mises stress, the ball and substrate were modeled only in the elastic mode to reduce the computation time. The contact pressure was also measured and found to increase with the decreasing mesh size in the contact region. In particular, the 0.05 mm and 0.025 mm mesh sizes showed much lower contact pressure values than the theoretical value. To obtain the theoretical value, the gradient mesh for the substrate was used, and a contact pressure value of 205.11 MPa (Table 4) was obtained, which was close to the contact pressure value calculated theoretically (±15%). In the gradient mesh, the mesh sizes were gradually reduced from edge to center from both sides and from bottom to top. Thus, the element size was made to be very small at the friction contact area. The gradient mesh provided an effect equal to that of the smaller mesh size model and reduced the computational time significantly. The von Mises stress values of the three mesh sizes were compared during sliding, and the maximum stress occurred in the middle of the contact region and spread around it. The von Mises stress was measured at the center of the substrate and coating surfaces, and the obtained values are given in Figure 5d. The von Mises stress values of the coating (152.39 MPa) and substrate (163.79 MPa) for the gradient mesh simulation were higher than the stress of the 0.025 mm and 0.05 mm mesh simulations. The von Mises stress of the coating was higher than the substrate, but the difference in the von Mises stress between the coating and substrate decreased with the decreasing mesh size. This was due to the reduction in the contact region, which led to an increase in the von Mises stress, as observed in Figure 5a–c. Contrary to the other two mesh sizes, the gradient mesh simulation showed a high von Mises stress on the substrate than the coating. The computation time increased with the decreasing mesh size.

### 3.6. Proliferation, Viability and Morphology of Preosteoblasts

#### 3.6.1. Cell Proliferation and Viability

**Figure 6:**
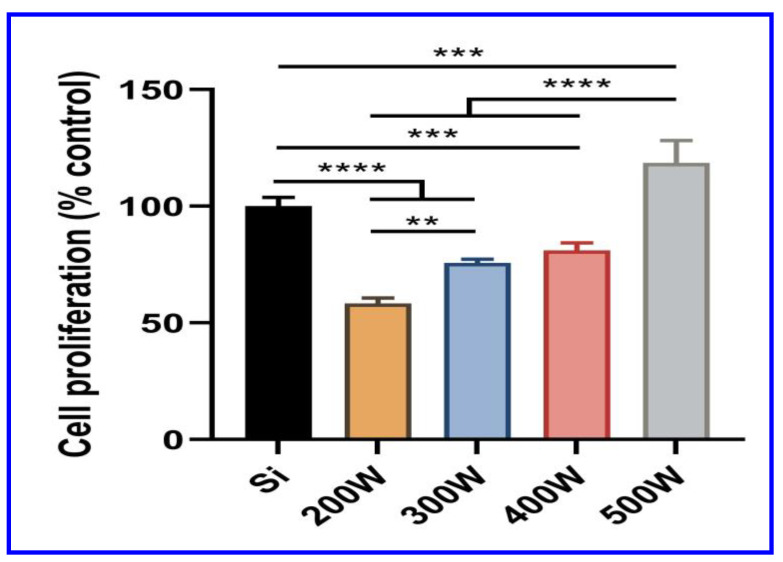
The percentage proliferation of preosteoblasts cultured on substrates was measured at day 3 and normalized to the control group. Error bars are means with standard deviations and the significant effects of different groups on bone regeneration are denoted as ** (*p* < 0.01), *** (*p* < 0.001) and **** (*p* < 0.0001), respectively.

**Figure 7:**
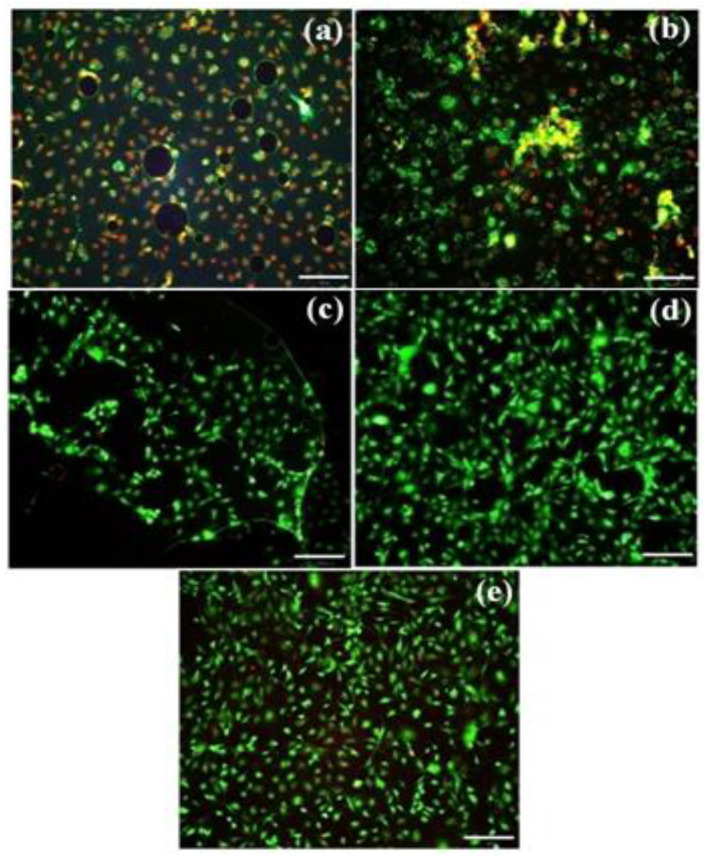
The viability assay of preosteoblasts on (**a**) uncoated Si and (**b**) 200 W, (**c**) 300 W, (**d**) 400 W and (**e**) 500 W substrates performed on day 3 (green: live cells; red: dead cells; scale bar = 200 μm).

Figure 6 shows the cell proliferation on the bare Si (100) substrate (control) and the a-C coatings on the Si (100) substrate. The percentage of cell proliferation on the a-C-coated Si (100) substrates were evaluated and compared with the bare Si (100) substrate (control). The percentages of cell growth were found to be 58.4%, 75.7%, 81.1% and 126.42% for 200 W, 300 W, 400 W and 500 W target powers, respectively. Among the different target powers, the 500 W coating showed an enhanced cell proliferation (Figure 6). Furthermore, the live and dead cells were visualized using a fluorescence microscope (CKX41- F32FL; Olympus, Tokyo, Japan). Figure 7a–e shows the fluorescence microscopic images of the preosteoblast cells cultured for 3 days on the silicon substrate with different target power coatings of 200, 300, 400 and 500 W. The coatings on the Si (100) substrate with an increased target power resulted in a better cell attachment for a 3-day culture period of the preosteoblast cells. The cell viability on the bare Si (100) substrate was found to be weaker than on the substrates with a-C coatings. The 200 W coating exhibited toxicity to the cells, but the level was comparatively less than that of the bare Si (100) substrate. This outcome was presumed to be due to the poor adhesion of the a-C coating with a target power of 200 W. The cell viability was moderately improved in the 300 W coating compared with the 200 W coating. The 400 W coating showed a better cell viability than the 200 W and 300 W coatings. However, the highest cell viability among all the coatings was noticed for the 500 W coating. The cell viability test results suggest that the 500 W coating had a better preosteoblast biocompatibility than the 200, 300 and 400 W coatings.

#### 3.6.2. Scanning Electron Microscope Images of Preosteoblasts

**Figure 8:**
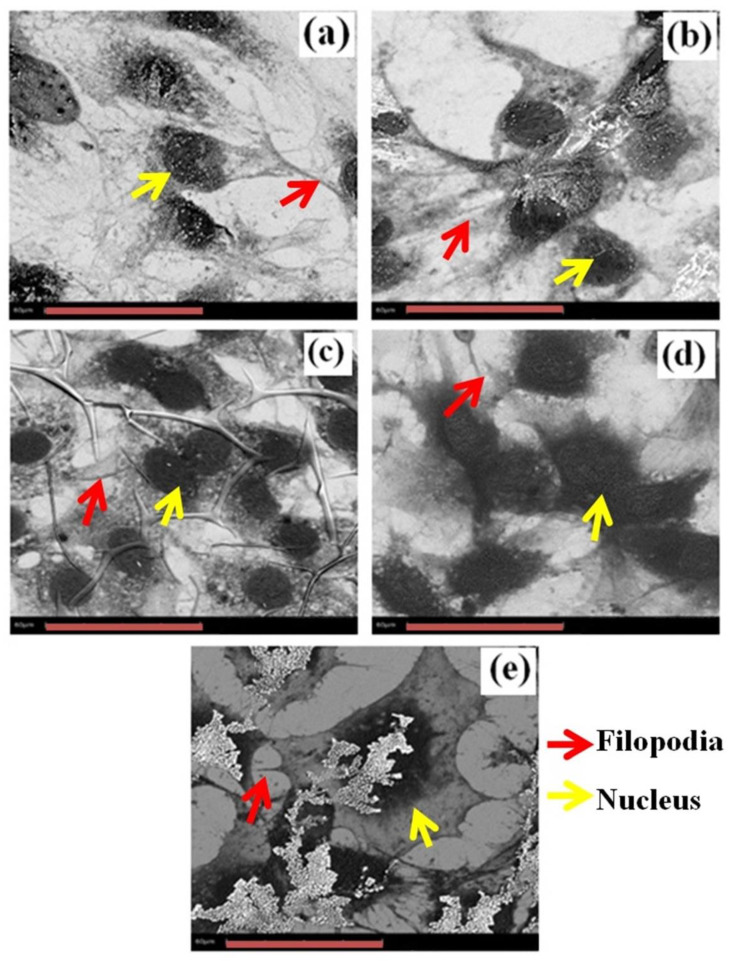
SEM image of preosteoblasts after 3-day incubation period on (**a**) uncoated Si and (**b**) 200 W, (**c**) 300 W, (**d**) 400 W and (**e**) 500 W coatings (scale bar = 60 μm).

The SEM images of the 3-day incubated preosteoblasts are shown in Figure 8a–e. A significant improvement in the cell adhesion to the substrates was noticed on day 3. The bare substrate showed poorer filopodia connection than the a-C-coated substrates. The interactions of filopodia between the cells improved for the coated substrates when compared to the bare substrate. It was also important to investigate the spreading profile of the cells on the various substrates to assess the biocompatibility of the samples. It was found that cell spreading and the filopodia interaction increased as the surface coating power increased. Thus, the results reveal that the a-C coatings with increased target power established an efficient and favorable platform that enhanced the interaction of the cells with the substrates. The coatings with higher target powers (500 W) resulted in an increased binding affinity between the substrate and the preosteoblast cells.

## 4. Discussion

Regarding the tribological properties, the 400 W coating showed the lowest COF and the highest wear resistance compared with all the other coatings. According to the AFM analysis, the 200 and 300 W coatings had uniform and smooth topographies, the 400 W coating had a relatively uniform topography, and the 500 W coating had an uneven topography. It should be noted that the 200 coating showed a relatively high wear rate, even though it had a smooth surface topography. This outcome was attributed to the increased adhesion between the smooth coating surface and the alumina sphere, as reported in previous works [48]. The 300 W coating showed a lower wear rate, but the film was found to be delaminated in some places along the wear track. The increases in the friction and wear rate of the 500 W coating were thought to be due to the high surface roughness and uneven surface topography [49]. It was also postulated that an increase in the contact angle decreases the friction; this might be due to the reduced friction and wear rate of the 400 W coating [50]. However, the friction and wear rates of the 300 W and 400 W coatings are smaller than those of the 200 W and 500 W coatings. The 400 W coating has a uniformly rough surface and a high contact angle, which leads to the lowest wear rate and friction among all the other coatings. The contact pressure obtained from the FEA with the lowest mesh size at the contact region was found to have a similar value to that of the mean theoretical value, whereas the other mesh sizes were found to have much lower values in comparison to the theoretical value. This confirms that the mesh size is very important in correlating the experimental and simulation results. The FEA showed a considerable increase in the von Mises stress with the decreasing mesh size. The maximum stress on the coating was higher than that on the substrate in all mesh sizes except for the gradient mesh simulation. This difference in the substrate and coating in all the simulations might be due to the mechanical property mismatch between the coating and substrate [51].

In the case of biocompatibility, the 500 W coating showed the highest cell growth rate than all the other coatings. The cell viability, proliferation and behavior of the preosteoblasts on the 400 and 500 W coatings were generally better compared with the other coatings. The results clearly show that the biocompatibility increased with the increasing target power during the sputtering process. The 400 and 500 W coatings offer higher cell growth capabilities, which suggest increased biocompatibility due to the strongly adhered carbon coating on the substrates. There are various reports [28,52] on the effect of surface roughness on the cell viability, but in the present study, the roughness values are nearly equal; hence, the effect of surface roughness on the preosteoblast cells on the prepared substrates can be negligible. The wettability of the substrates also plays an important role in cell growth. Even though the wettability of the coating changed a after few hours in the cell medium, the protein affinity on the substrate was high on the hydrophobic surface, which enhanced the cell adhesion [28,29]. It was reported that the protein affinity, which is important for cell adhesion, is higher for hydrophobic surfaces than for hydrophilic surfaces [29,53]. In the present study, the wettability of the coatings prepared at 200 and 300 W were hydrophilic, and the coatings prepared at 400 and 500 W were hydrophobic. Thus, the initial protein affinity was thought to be higher for the 400 and 500 W coatings than for the 200 and 300 W coatings, which led to better cell proliferation on the 400 and 500 W coatings [52].

## 5. Conclusions

The a-C coatings were deposited on Si (100) substrates via the RF magnetron sputtering technique with different graphite target powers. The characteristics of the coatings were investigated using a Raman spectrometer, AFM, and a contact angle measurement system. The tribological behavior and the preosteoblast cell biocompatibility of the coatings were also analyzed. The lower shift of the G band with the increasing graphite target power was determined via Raman analysis, which confirms the increase in the *sp^3^* bonds in the a-C coatings. The 500 W coating had the highest surface roughness and an uneven topography. The contact angle measurement studies indicated the transformation of the coatings from hydrophilic to hydrophobic as the target power increased. The friction and wear properties of the 300 and 400 W coatings were found to be better than the other coatings. Particularly, the uniformity of the surface roughness and hydrophobic nature of the 400 W coating led to low friction and wear rates. The Finite Element Analysis (FEA) of the 400 W coating confirms that lowering the mesh size enhances the von Mises stress, and particularly, the contact pressure approaches the theoretical value, which confirms the importance of mesh size in friction simulation. The cell proliferation was found to be the highest on the 400 and 500 W coatings than on all the other coatings. Based on the overall tribological properties and the biocompatibility assessment of the a-C coatings, 400 W was identified to be the optimum target power.

## Figures and Tables

**Table 1:** Friction test parameters.

Load	Counter Tip	Stoke Length	Temperature	Relative Humidity	Sliding Speed
10 mN	1 mm diameter Al_2_O_3_ ball	4 mm	Room temperature	40%	2 mm/s

**Table 2:** Raman peak parameters and AFM analysis of a-C coatings prepared at different target powers.

Sample	D Peak Position cm^−1^	G Peak Position cm^−1^	R_a_ (nm)	R_z_ (nm)	R_q_ (nm)	R_pv_ (nm)
200 W	1410	1582	1.32	9.03	1.74	12.42
300 W	1406	1581	0.99	4.57	1.02	6.59
400 W	1401	1578	1.24	5.90	1.31	11.33
500 W	1378	1572	3.3	20.4	4.20	21.82

**Table 3:** Average coefficient of friction (CoF) and wear rate of a-C coatings with different target powers.

Sample	Average CoF	Wear Rate (10^−7^ mm^3^/N*mm)
200 W	0.50	1.35
300 W	0.49	0.47
400 W	0.45	0.54
500 W	0.71	5.58

**Table 4:** Details and findings from FEA simulation.

Substrate Mesh Size (mm)	Coating Element Type	Substrate Element Type	Counter Tip Element Type	Contact Pressure (MPa)	Computation Time (Minutes)
0.05	S4R	C3D8R	C3D10	2.90	13
0.025	S4R	C3D8R	C3D10	8.05	154
Gradient	S4R	C3D8R	C3D10	205.11	1152

## Data Availability

Not applicable.

## References

[1] Berman D., Erdemir A., Zinovev A.V., Sumant A.V. (2015). Nanoscale friction properties of graphene and graphene oxide. Diam. Relat. Mater..

[2] Yang X., Zou T., Shi C., Liu E., He C., Zhao N. (2016). Effect pf carbon nanotube (CNT) content on the properties of in-situ synthesis CNT reinforced Al composites. Mater. Sci. Eng. A.

[3] Bae K.M., Yang H.D., Tufa L.T., Kang T.J. (2015). Thermobattery based on CNT coated carbon textile and thermoelectric Electrolyte. Int. Precis. J. Eng. Manuf..

[4] Zhao S., Zheng Z., Huang Z., Dong S., Luo P., Zhang Z., Wang Y. (2016). Cu matrix composites reinforced with alighed carbon nanotubes: Mechanical, electrical and termal properties. Mater. Sci. Eng. A.

[5] Ryu H.J., Kim S.H., Hong S.H. (2000). Effect of deposition pressure on bonding nature in hydrogenated amorphous carbon films processed by electron cyclotron resonance plasma enhanced chemical vapor deposition. Mater. Sci. Eng. A.

[6] Fu Q.-G., Tan B.-Y., Zhuang L., Jing J.-Y. (2016). Significant improvement of mechanical properties of carbon/carbon composites by in situ growth of SiC nanowires. Mater. Sci. Eng. A.

[7] Hong Y.-S., Lee S.-R., Kim J.-H. (2015). Application of a DLC-Coating for improving hydrostatic piston shoe bearing performance under mixed friction conditions. Int. Precis. J. Eng. Manuf..

[8] Lungu C.P. (2005). Nanostructure influence on DLC-Ag tribological coatings. Surf. Coat. Technol..

[9] Yari M., Larijani M.M., Afshar A., Eshghabadi M., Shokouhy A. (2012). Physical properties of sputtered amorphous carbon coatings. J. Alloys Compd..

[10] Cui F.Z., Li D.J. (2000). A review of investigations on biocompatibility of diamond-like carbon and carbon nitride films. Surf. Coat. Technol..

[11] Feng B., Cao D.M., Meng W.J., Xu J., Tittsworth R.C., Rehn L.E., Baldo P.M., Doll G.L. (2001). Characterization and mictrostructure and mechanical behavior of sputter deposited Ti-containing amorphous carbon coatings. Surf. Coat. Technol..

[12] Quinn J., Lemoine P., Maguire P., McLaughlin J. (2004). McLaughlin. Ultra-thin tetrahedral amorphous carbon films with strong adhesion, as measured by nanoscratch testing. Diam. Relat. Mater..

[13] An W., Zhao X., Gu L., Su R. (2014). Nanomechanical properties and surface wettability of carbon films prepared by magnetron sputtering. Key Eng. Mater..

[14] Lam B.X. (2008). Diamond—Like carbon coatings for tribological applications. Sci. Technol. Dev..

[15] Charitidis C.A. (2010). Nanomechanical and nanotribological properties of carbon-based thin films: A review. Int. Refract. J. Met. Hard Mater..

[16] Afshar A., Yari M., Larijani M.M., Eshghabadi M. (2010). Effect of substrate temperature on structural properties and corrosion resistance of carbon thin films used as bipolar plates in polymer electrolyte membrane fuel cells. Alloy J. Compd..

[17] Svahn F., K-Asa R., Wallen E. (2003). The influence of surface roughness on friction and wear of machine element coatings. Wear..

[18] Liu D., Benstetter G., Lodermeier E. (2003). Surface roughness, mechanical and tribological properties of ultrathin tetrahedral amorphous carbon coatings from atomic force measurements. Thin Solid Film..

[19] Li Q., Li W., Feng Q., Wang P., Mao M., Liu J., Zhou L., Wang H., Yao H. (2014). Thickness-depentant fracture of amorphous carbon coating on SnO^2^ nanowire electrodes. Carbon.

[20] Bhushan B. (1999). Chemical, mechanical and tribological characterization of ultra-thin and hard amorphous carbon coatings as thin as 3.5: Recent developments. Diam. Relat. Mater..

[21] Chang Y.-Y., Huang H.-L., Chen Y.-C., Hsu J.-T., Shieh T.-M., Tsai M.-T. (2014). Biological characteristics of the MG-63 Human Osteosarcoma cells on composite Tantalum Carbide/ Amorphous Carbon Films. PLoS ONE.

[22] Lukyanchenko V., Donkov N., Zykova A., Safonov V., Miroshnichenko K. (2015). In vitro biocompatibility of amorphous carbon based coatings by varying of surface chemistry and nitrogen concentrations. Probl. At. Sci. Technol..

[23] Dearnaley G., Arps J.H. (2005). Biomedical applications of diamond-like carbon (DLC coatings): A review. Surf. Coat. Technol..

[24] Subbiah R., Du P., Van S.Y., Suhaeri M., Hwang M.P., Lee K., Park K. (2014). Fibronectin-tethered graphene oxide as an artificial matrix for osteogenesis. Biomed. Mater..

[25] Dhandapani V.S., Subbiah R., Elangovan T., Madhankumar A., Park K., Gasem Z.M., Veeravazhuthi V., D-Kim E. (2016). Tribological properties, corrosion resistance and biocompatibility of magnetron sputtered titanium-amorphous carbon coatings. Appl. Surf. Sci..

[26] Rodil S.E., Olivares R., Arzate H., Muhl S. (2003). Properties of carbon films and their biocompatibility using in-vitro tests. Diam. Relat. Mater..

[27] Jiang C.-P., Chen Y.-Y. (2014). Biofabrication of Hybrid Bone Scaffolds using a dual nozzle bioplotter and in-vitro study of Osteoblast cell. Int. Precis. J. Eng. Manuf..

[28] Chai F., Mathis N., Blanchemain N., Meunier C., Hildebrand H.F. (2008). Osteoblast interaction with DLC-coated Si substrates. Acta Biomater..

[29] Randeniya L.K., Bendavid A., Martin P.J., Amin M.S., Rohanizadeh R., Tang F., Cairney J.M. (2010). Thin-film nanocomposites of diamond-like carbon and titanium oxide; Osteoblast adhesion and surface properties. Diam. Relat. Mater..

[30] Penkov O.V., Pukha V.E., Starikova S.L., Khadem M., Starikov V.V., Maleev M.V., Kim E.-D. (2016). Highly wear-resistant and biocompatible carbon nanocomposite coatings for dental implants. Biomaterials.

[31] Dhandapani V.S., Elangovan T., Madhankumar A., Shin K.S., Veeravazhuthi V., Yau S.Y., Kim C.L., D-Kim E. (2014). Effect of Ag content on the microstructure, tribological and corrosion properties of amorphous carbon coatings on 316L SS. Surf. Coatings Technol..

[32] Archard J.F. (1961). Single Contacts and Multiple Encounters. Appl. J. Phys..

[33] Shon I.J., Wang H., Cho S.W., Kim W. (2011). Mechanical Synthesis and Rapid Consolidation of Nanocrystalline TiAl-Al2O3 Composites by High Frequency Induction Heated Sintering. Mater. Trans..

[34] Bhushan B., Li X. (1997). Micromechanical and tribological characterization of doped single-crystal silicon and polysilicon films for microelectromechanical systems devices. Mater. J. Res..

[35] https://www.sonelastic.com/en/fundamentals/tables-of-materials-properties/ceramics.html.

[36] Dolbow J., Gosz M. (1996). Effect of out-of-plane properties of a polyimide film on the stress fields in microelectronic structures. Mech. Mater..

[37] Thangavel E., Ramasundaram S., Pitchaimuthu S., Hong S.W., Lee S.Y., Yoo S.S., Kim D.E., Ito E., Kang Y.S. (2014). Structural and tribological characteristics of poly(vinylidene fluoride)/functionalized graphene oxide nanocomposite thin films. Compos. Sci. Technol..

[38] Ishpal I., Panwar O., Srivastava A., Kumar S., Tripathi R., Kumar M., Singh S. (2011). Effect of substrate bias in amorphous carbon films having embedded nanocrystallites. Surf. Coat. Technol..

[39] Pradhan D., Sharon M. (2007). Opto-electrical properties of amorphous carbon thin film deposited from natural precursor camphor. Appl. Surf. Sci..

[40] Wang Y., Li H., Ji L., Liu X., Wu Y., Lv Y., Fu Y., Zhou H., Chen J. (2012). Synthesis and characterization of titanium-containing graphite-like carbon films with low internal stress and superior tribological properties. Phys. J. D Appl. Phys..

[41] Fadzilah A.N., Dayana K., Rusop M. (2013). Fabrication and characterization of camphor based amorphous carbon thin films. Procedia Eng..

[42] Wei Q., Sharma A.K., Sankar J., Narayan J. (1999). Mechanical properties of diamond-like carbon composite thin films prepared by pulsed laser deposition. Compos. Part B.

[43] Asl A.M., Kameli P., Ranjbar M., Salamati H., Jannesari M. (2015). Correlation between microstructure and hydrophobicity properties of pulsed laser deposited diamond-like carbon films. Superlattices Microstruct..

[44] Choi H.W., Dauskardt R.H., Lee S.-C., Lee K.-R., Oh K.H. (2008). Characteristic of silver doped DLC films on surface properties and protein adsorption. Diam. Relat. Mater..

[45] Schlebrowski T., Beucher L., Bazzi H., Hahn B., Wehner S., Fischer C.B. (2019). Changing Contents of Carbon Hybridizations in Amorphous Hydrogenated Carbon Layers (a-C:H) on Sustainable Polyhydroxybutyrate (PHB) Exhibit a Significant Deterioration in Stability, Depending on Thickness. J. Carbon Res..

[46] Tay B.K., Sheeja D., Lau S., Guo J. (2003). GuoStudy of surface energy of tetrahedral amorphous carbon films modified in various gas plasma. Diam. Relat. Mater..

[47] Schulz H., Leonhardt M., Scheibe J.H., Schultrich B. (2005). Ultra hydrophobic wetting behaviour of amorphous carbon films. Surf. Coat. Technol..

[48] Kakas D., Terek P., Miletic A., Kovacevic L., Vilotic M., Skoric B., Krumes D. (2013). Friction and wear of low temperature deposited TiN coating sliding in dry conditions at various speeds. Tehn. Vjesn..

[49] Riyadh A.A.S., Haftirman, Khairel A., Yarub A.D. (2012). The influence of roughness on the wear and friction coefficient under dry and lubricated sliding. Int. Sci. J. Eng. Res..

[50] Zhang C., Fujii M. (2015). Influence of wettability and mechanical properties of tribological performance of DLC coatings under water lubrication. Surf. J. Eng. Mater. Adv. Technol..

[51] Khadem M., Penkov O.V., Jais J., Bae S.M., Dhandapani V.S., Kang B., Kim D.E. (2021). Formation of discrete periodic nanolayered coatings through tailoring of nanointerfaces—Toward zero macroscale wear. Sci. Adv..

[52] Akasaka T., Yokoyama A., Matsuoka M., Hashimoto T., Watari F. (2010). Thin films of single-walled carbon nanotubes promote human osteoblastic cells (Saos-2) proliferation in low serum concentrations. Mater. Sci. Eng. C.

[53] Feng B., Weng J., Yang B.C., Qu S.X., Zhang X.D. (2003). Characterization of surface oxide films on titanium and adhesion of osteoblast. Biomaterials.

